# FROM SLICES TO SPACES Design ideation on architectural models through AI-generated image sequences

**DOI:** 10.1177/14780771251352951

**Published:** 2025-07-07

**Authors:** Mathias Bank, Johannes Schlusche, Shervin Rasoulzadeh, Stefan Rutzinger, Kristina Schinegger

**Affiliations:** 1 Department of Design, i.sd | Structure and Design, University of Innsbruck, Innsbruck, Austria; 2 Center for Geometry and Computational Design, Vienna University of Technology, Wien, Austria

**Keywords:** AI, architectural design, concept model, form finding, ideation, video diffusion

## Abstract

The paper presents a novel methodology for applying AI-driven style transfer to complex 3D architectural models. By converting 3D models into 2D image sequences, the process integrates sequential slicing, training, video-guided diffusion and reconstruction to transform existing 3D models based on text, image, or video prompts into new stylised forms. This enables architects to explore diverse design concepts, focusing on spatial composition, visual appearance and tectonics through high-resolution outputs that capture both exterior and interior spatial relations. The results demonstrates the setups potential in enhancing early-stage design ideation through AI, by both outperforming existing video diffusion platform while also facilitating a fast exploration of different outcomes - capabilities which were validated in a design course. The study highlights an approach for utilising advanced 2D image-based AI models to generate intricate and meaningful 3D architectural transformations.

## Introduction

The progress of architectural design is intimately linked to technological advancements, and the arrival of Artificial Intelligence (AI) is a classic example of this dynamic. AI has, in recent years, become a major influence in the field, offering new methodologies that might redefine and reinvent conventional design paradigms - particularly through the adoption of Artificial Neural Networks (ANNs).^1^ Among these advancements, text-to-image generative models have become increasingly popular, providing architects in the early phases of a design process with new opportunities to swiftly and effectively visualise ideas.^2,3^

A central objective in AI research is to develop multimodal neural networks capable of interpreting, synthesising, and connecting information across different modalities. The 2021 release of OpenAI’s CLIP (Contrastive Language–Image Pre-training), a system that can effectively process visual ideas from natural language inputs, was a major milestone towards this objective.^4^ Its integration with image-centric generative approaches from Generative Adversarial Networks (GANs) to Diffusion Probabilistic models further expanded its functionality, leading to the rise of platforms such as Midjourney, DALL ⋅ E, and Stable Diffusion. This has enabled the development of multimodal networks that can generate images derived from textual prompts.^5,6^ These networks provide alternative ways to engage with design challenges through the generation of synthetic data spaces, offering architects a unique lens to explore and evaluate outcomes, while providing new stylistic impulses for design.

### Research topic

The concept of ‘style’ in architecture has historically oscillated between two opposite ends: it is either viewed as a distraction from the functional, technological or social dimensions of building, or it is seen as a primary method of cultural and artistic expression. On one hand, critics such as Hermann Muthesius and Sigfried Giedion approached style with caution, arguing that an excessive focus on ornamentation could obscure more pressing architectural concerns.^7,8^ On the other hand, theorists like Walter Curt Behrendt identified style as a unifying concept fundamental to the field’s theoretical underpinnings.^9^ Traditionally, buildings were categorised into styles like ‘Renaissance’ or ‘Baroque’ based on recurring characteristics that extended beyond simple visual elements - encompassing proportions, spatial hierarchies and larger cultural frameworks.^10^ Recent developments in machine vision and AI-driven image processing, especially through ANNs, have triggered a renewed interest and changed our understanding of style.^11^ These algorithms mimic human visual processes to analyse vast datasets of images, extracting distinctive visual patterns without relying on predefined parameters or human biases. This data-driven perspective on style is shaped by the statistical distributions of visual data, defined by pixels, edges and textures, enabling image-based AI-networks to recognise, reinterpret and generate styles beyond the boundaries of established historical classifications.

One of the most prominent creative techniques within this context is style transfer which enable ANNs to blend and recombine different aesthetic languages.^12^ The process involves extracting stylistic information from one set of data and applying it to another, facilitating a cross-fertilisation of visual characteristics, providing designers with a method to combine different creative concepts. This is especially relevant for conceptual exploration during the early design phase, where designers often iterate through different stylistic options before converging on a final design.^13^ Given that most of the current implementations revolve around image-based setups, architectural drawings have emerged as a central medium for exploring their potential within an architectural context.^14,15^ Compared with other image-based mediums such as sketches, diagrams or photos, technical drawings provide a precise and standardised depiction of design parameters, making them an ideal medium for exploring design ideas through ANNs.^16^

Plans and sections, the two main architectural drawings, offer a similar representation between mass and space/void. Whereas plans mostly depict horizontal slices through walls and not floors, sections have the versatility to illustrate cuts encompassing both walls and floors, while organising space in relation to tectonics and material systems.^17^ Greg Lynn’s concept of serial transsections, discussed in *Probable Geometries*, extends these traditional methods by presenting a sequence of planar slices that collectively convey a precise portrayal of three-dimensional structures.^18^ This approach parallels tomographic imaging, where a succession of cross-sectional images can be compiled to interpret volumetric information.

From a computational perspective, this slicing procedure can be used to generate training datasets for ANNs, each slice depicting localised spatial information relating to structural features, organisational concepts or other metadata. Moreover, new developments with video and animations through Text-to-Image diffusion models have enabled the application and development of stylistic transformations across successive frames,^19,20^ introducing new opportunity for design exploration. By treating a ‘tomography’ of a three-dimensional structure as equivalent to frames in a video, it therefore becomes possible to stylistically experiment with entire 3D models along their vertical or horizontal dimension through modern image-based ANNs.

### Related work

Though the realm of style transfer has been examined in architectural design,^21–23^ it’s often been limited to singular images, with its potential on intricate 3D models remaining largely unexplored.

Other research projects have explored a similar 3D to 2D encoding in order to apply image-driven ANNs on 3D models. In the work by Zhang and Blasetti^24^ various applications of GANs are used to create stylistic hybrids between existing models through sequential image operations. The approach also utilizes a sequential section operation to encode 3D models into the 2D domain and shows promising results by applying the style of an existing 3D model to another. The authors of L*earning Spatiality - A GAN method for designing architectural models through labelled sections*^25^ further expand on this approach by including semantic data through coloured images, while demonstrating its ideation potential within a design seminar. However, both approaches struggle with traces of uniaxial slicing, which relates to the inability of the setup to coherently apply a style across the entire image stack in their generated outcomes. In a related project by Zhang and Huang,^26^ the authors try to tackle this issue by stitching a stack of 64 sequential slices into a single image, organized in an 8 × 8 grid. Enabling the networks to see the entire 3D model through one image simplifies the style-transfer process, but also limits the resolution of each slice, resulting in less detailed outcomes. Along a similar vein, the authors of “Collapsing Complexity”^27^ provide a novel encoding method for complex 3D models. By utilizing the concept behind vertex animation textures, they translate each section into a row of pixels through a point cloud notation. This results in an incredibly efficient encoding which encompasses 1024 slices of a model within a single 1024 × 1024 image. Though the encoding achieves an impressive 3D model resolution, the image itself is indecipherable to a human, and as such ANN based operations are harder to evaluate, optimize and use, resulting in a high degree of noise in their generated outcomes.

A more promising approach for exploring style transfer on architectural models relates to the rapidly evolving field of Text-to-3D generation. Thanks to the immense success of generative image models such as diffusion models, a workaround has been discovered to utilize their large aligned text-image datasets to generate novel 3D models. This is achieved through a through a 3D-aware image synthesis process focusing on generating images that encompass an understanding of structures and spatial relationships within a scene.^28^ Compared with 2D image synthesis, which is limited to flat planes, the technique uses 3D representations such as meshes, voxel or Neural Radiance Fields^29^ to store the synthesised spatiality from the 2D image. Upon this foundation, DreamFusion,^30^ HiFA^31^ and Style-NeRF2NeRF^32^ are three of many exciting steps towards good Text-to-3D generation. In his contribution to Diffusions in Architecture,^33^ Immanuel Koh explores how these developments can be integrated into creative processes. Within the context of architectural design, this has the potential to streamline the design process and skip the intermediate step of translating 2D images into 3D forms. This would enable a fast exploration of a significantly broader range of design ideas. However, the current state of this method offers only low-resolution boundary shapes with no interior qualities, making it unideal for exploring complex architectural style transfers.

### Goal and objectives

In this paper, we explore and test how image sequences derived from architectural models can be combined with recent advances in AI diffusion and video creation to establish a novel workflow for text-, image- and video-based style transfer on complex 3D models. By applying a sequentially aware deformation process, the objective is to transform the source model toward a chosen stylistic goal. This enables designers to rapidly explore a range of design concepts relating to the overall spatial composition, visual appearance and tectonics of the model, offering a variety of fast design iterations for enhancing the early design stages.

The approach tackles several current issues: it addresses the limited exploration of style-transfer techniques on intricate 3D architectural models, resolving spatial discontinuities common in existing image-based methods. Additionally, it responds to the underexplored potential of generative AI tools in early-stage 3D design ideation, introducing an efficient workflow for rapidly exploring diverse stylistic transformations - demonstrated through a proof-of-concept implementation within a design seminar.

### Relation to prior work

While our eCAADe 2024 conference paper *’Diffused Tomography: Design ideation on architectural models through image sequences’*^34^ introduced the basic tomographic slicing + video-diffusion idea, this manuscript extends the former by expanding the proposed framework, adding additional results, further discussing its application while providing new insights throughout. Specifically, three novel contributions are added to address shortcomings in the eCAADe version:

• Expansion of the workflow and the results to include image- and video-prompts beyond the initial text-prompts to better showcase its ideation versatility.
• Comparison of the proposed video-diffusion workflow against existing pipelines from RunwayML’s GEN-3 and OpenAI’s Sora to qualitatively document and assess its novelty.
• Two in-depth case studies from a bachelor design seminar, showing how this method can function as an ideation tool within a design context.

## Methodology

**Figure 1.**
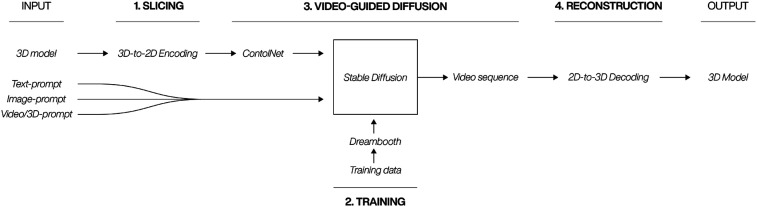
Four-stage workflow for 3D to 2D encoding, diffusion, and reconstruction.

The proposed framework for integrating two-dimensional image diffusion models with pre-existing 3D architectural models consists of four key elements: Slicing, training, video-guided diffusion, and reconstruction. Figure 1 depicts this process and commences with 3D model and either a text-, image- or video-based prompt serving as inputs. The culmination of this procedure is the generation of an enhanced 3D model, which has been stylistically transformed by the initial inputs. This new approach facilitates the application of style transfer from text, image or video to intricate 3D models and has the potential to enhance and accelerate the exploration of design concepts through ANNs.

### Slicing

The purpose of the slicing is to translate information within a 3D model to 2D images, so the 3D data can be used in connection with existing 2D image diffusion workflows. Inspired by the mentioned related work of Zhang and Blasetti,^24^ and later work by Bank et al.,^25^ the encoding leverages a sequential slicing operation of the 3D model similar to the process of computed tomography. This technique produces a sequence of images that depict the interaction between solid and empty areas in a 3D model. Captured at regular intervals along a specific slicing direction, these images can also convey additional details beyond the mass-void relationship. By utilizing colour, they can provide further insights into aspects like material properties or the model’s functional program.

**Figure 2.**
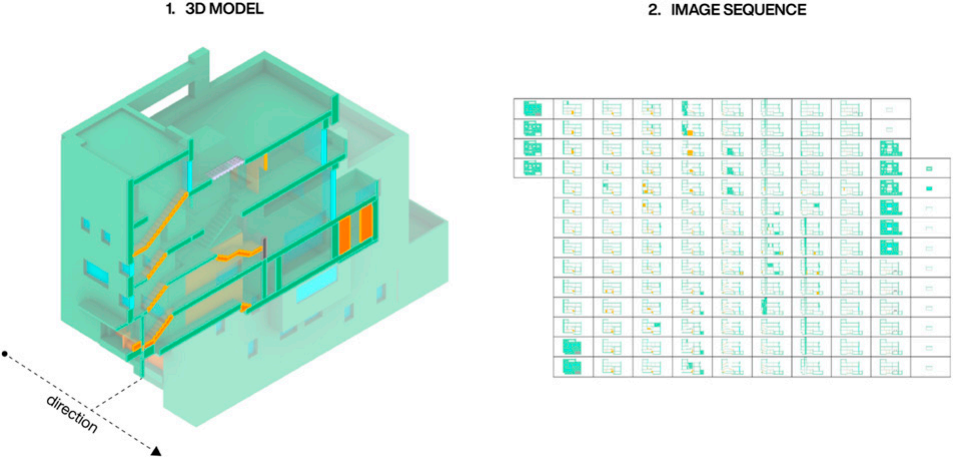
A 3D model depicted with its corresponding image sequence.

To evaluate the system, we selected a consistent image resolution of 1024 × 512 pixels and a slicing interval of 10 cm, tailored to suit the specific 3D dataset used (see Figure 5). We opted for a horizontal slicing direction, as this orientation produces images similar to architectural sections. In contrast to plans, this slicing direction includes both cut walls, floors and ceilings, heights of spaces, and better demonstrates how space is organized with respect to structural design and material systems. Furthermore, colours were used to store the material labels from the utilized dataset within the image sequences (see Figure 2).

### Training

The setup relies on existing image diffusion models, which have been trained and optimized by Stability AI. In our case, the SDXL-Turbo model^35^ is used since it allows for high quality at low sampling counts. As these models are general text-to-image models, we utilize Dreambooth^36^ to fine-tune the model checkpoints with our own images. This enables us to teach the diffusion model the visual semantics of our encoding process, while still benefitting from the capabilities of the existing model.

The training was done once for the utilized dataset and would only need to be redone if input architectural 3D models start to differ significantly from the existing dataset in either scale or labelling. From the specific dataset (see Fig. 5), 200 individual slices were used to train the visual style to the SDXL-Turbo model through Dreambooth. The trained visual style is activated using a designated unique key phrase, which is added to every text-, image- or video-prompt with a high priority to bring its effect to the forefront. For simplicities sake, the unique key phrase is not listed in any of the displayed prompts in this paper.

### Video-guided diffusion

At its core video-guided diffusion is similar to image-guided diffusion and typically works by merging a text prompt and an image into a unified vector. This fusion creates a guide for the diffusion process. The process towards the final image begins with a random seed, essentially a starting point filled with random noise. During the iterative denoising phase, the algorithm systematically reduces this noise, refining the image step by step - guided by the information encoded in the combined text and image vector. This progression ideally continues until the noise is sufficiently diminished, resulting in a final image that visually corresponds with the themes and styles dictated by the initial text prompt and image.^37^

Where video-guided diffusion differs, is that it aims to generate not only a single image but an entire sequence of images. To successfully do this, a certain amount of coherency between each image and its neighbours within the sequence is important. This is typically achieved by using a fixed seed for the entire image sequence while also integrating the appearance of the previous image into the unified vector that guides the creation of the current image.^20^ Several open-source community projects have over the last couple of years made this easily accessible through interfaces such as Stable Diffusion WebUI by AUTOMATIC1111,^38^ Deforum^39^ and many others.

**Figure 3.**
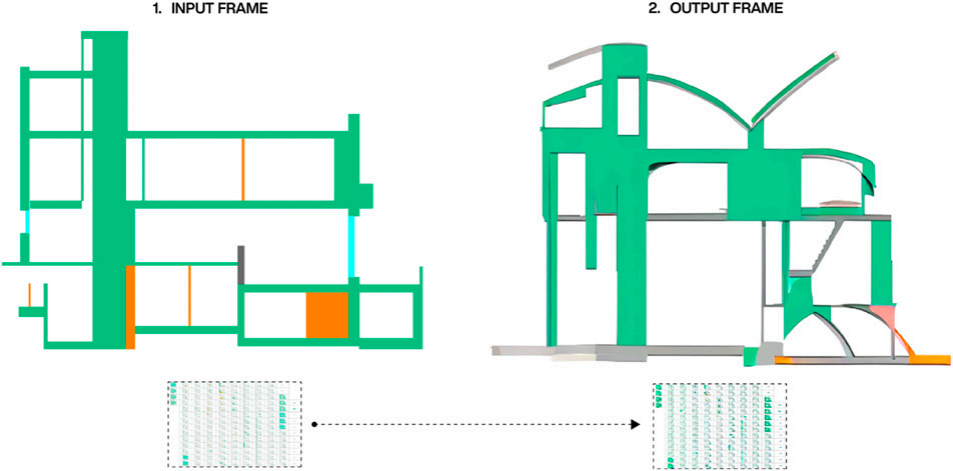
Input and output frame from the video-guided diffusion process. The used text-prompt is: architecture drawing inspired by suprematism and cubism.

Since our 3D to 2D encoding converts an architectural 3D model into an image sequence, we apply the principles within video-guided diffusion to sequentially deform it based on a text prompt. By utilizing ControlNet,^40^ we can apply filters such as segmentation or edge detection to our input image sequence, and combine this information with a text prompt to generate a unified vector that updates for every image. As a result, the video-guided diffusion process can use the characteristics of the input 3D model as a starting point, for constructing a new image sequence that corresponds to the text prompt (see Figure 3).

For our setup, we utilized Stable Diffusion WebUi together with Deforum^39^ and ControlNet. A fixed seed was used for each sequence, with seven steps for each image, a strength schedule of 0.3, and a classifier-free guidance scale (CFG) of 5, attempting a balance between speed, consistency across frames and vector strength. For ControlNet, we applied a segmentation model with a weight of 2. The remaining controls were left at their default values.

### Reconstruction

**Figure 4.**
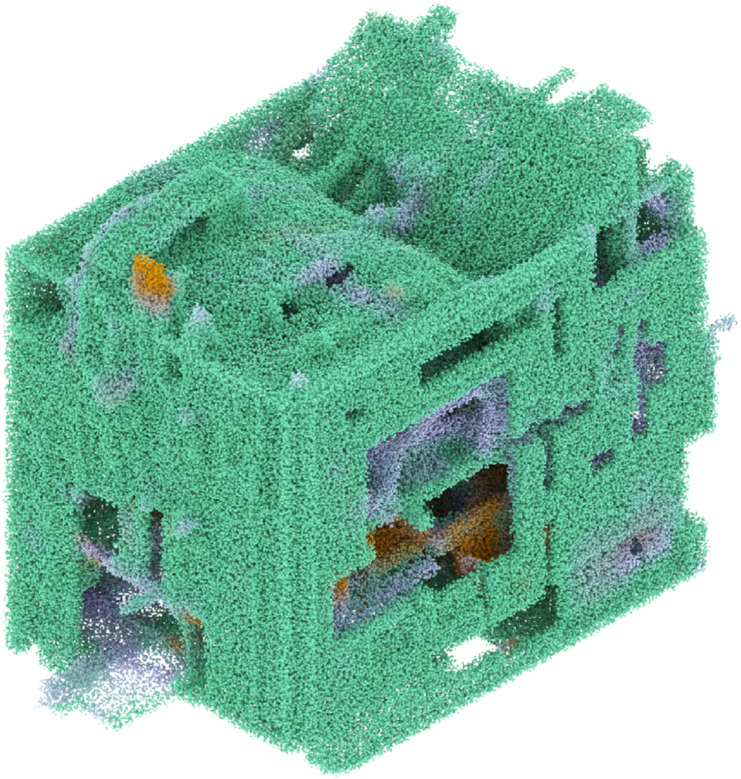
A concept cloud assembled through a generated image sequence. The reconstruction uses a point cloud based notation to transfer pixel into coloured points, reversing the slicing process.

To transform image sequences generated via video-guided diffusion into a 3D model, we utilize a point cloud technique. The process starts by sampling each image into a grid of points at a selectable resolution. These points inherit the colours from their corresponding locations in the images, while any points associated with unlabelled regions are discarded. The colours are as a final step remapped to the colour of the nearest material label to clean up smaller colour variances. We then reconstruct the sequence in reverse along the slicing vector, effectively reversing the 3D to 2D encoding. The resulting 3D point cloud (see Figure 4) is seen as a concept cloud, a spectral entity with an adjustable resolution that communicates spatial relationships between fragments of data. These concept clouds communicate new rhythms of mass, void and colour labels (in our setup material distribution) guided by the text prompt and the input 3D model.

## Results

**Figure 5.**
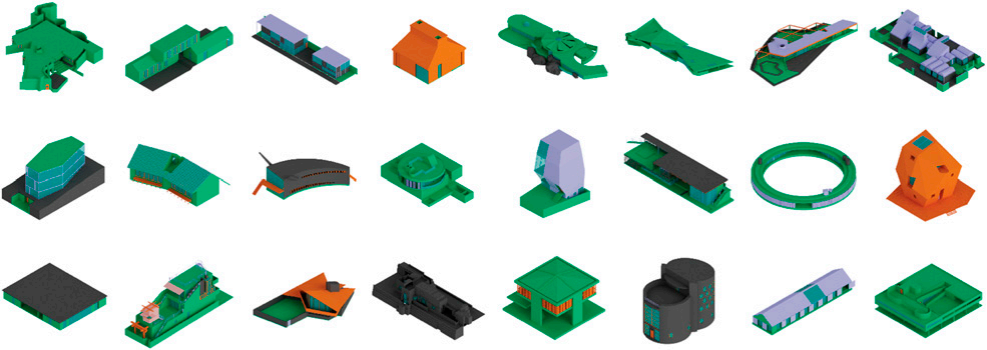
Subset of the 3D house models within the ArchHouse dataset. Each colour references a material: concrete (green), wood (orange), stone (grey), glass (cyan), metal (light purple) and other (salmon).

To test the framework, we utilized a collection of 150 labelled 3D models from the ArchHouse dataset^41^ (see Figure 5). These models represent canonical architectural houses, covering 500 years of architectural history. Each model was created using available published resources and labelled using a consistent colour scheme that represents their material characteristics. This was achieved by assigning specific colours to spatially enclosed volumes, effectively illustrating the materials’ appearance. Additionally, each model possesses a comparable level of detail and includes a fully modelled interior.

The workflows effectiveness is analysed through three main steps. First, the outcome from different prompts are shown to compare and evaluate their effectiveness in achieving precise stylistic changes in the input models. Secondly, the presented workflow is compared with existing video diffusion frameworks from RunwayML^42^ and OpenAI.^43^ Thirdly, the workflow is applied within the context of a bachelor design studio, demonstrating its potential as an ideation source during the early design stages.

### Evaluation

The evaluation explores the frameworks potential across three different types of prompts, text-, image- and video-based prompts. While the text- and image-based prompts acts as an interface to transform the image-sequence from the input model towards a targeted stylistic direction, the video-based prompt allows for the mutation of two 3D models. As the workflow encodes 3D models into a sequences of images, video-based prompts enable the input model to be directed towards the characteristics of another model, enabling an indirect 3D-to-3D style transfer.

#### Text-prompts

**Figure 6.**
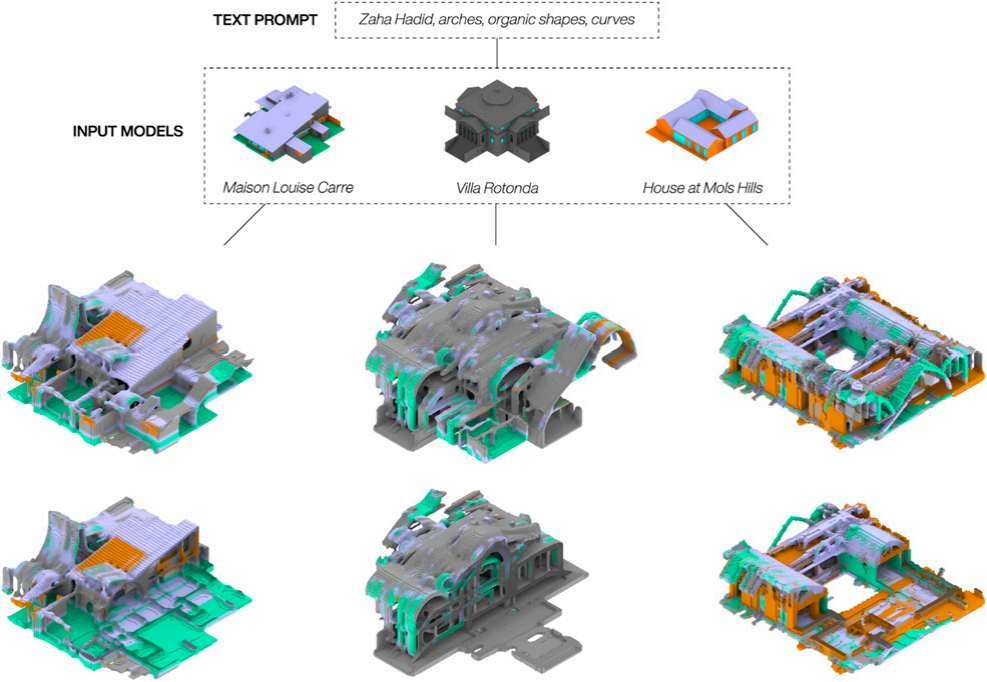
Text-prompts results: The same prompt with different input models.

We used two different strategies to evaluate the outcomes of the text-based prompts. For the first approach, the same prompt was combined with different input 3D models from the housing dataset. Here the goal was to deform very different houses with the same text-prompt to see if shared spatial characteristics would appear among the generated 3D models. Figure 6 highlights three results from this approach, visualizing both the whole and a cutout model to document the interior-exterior relations for each outcome. We used the text prompt Zaha Hadid, arches, organic shapes, curves, to create models with a more organic and fluid appearance. As seen in the figure, all the three generated models show a noticeable transformation towards an interpretation of the text prompt. Where Villa Rotonda was infused with more vaults and arches, Maison Louise Carre especially has a tower appearing not unlike a ceiling feature found in Herning Utzon House – a house which was also part of the training data. For the House at Mols Hills the pitched roofs were partially rounded, and two of them made into what could be interpreted as inhabitable roofs. A more blobby appearance within the interior organisation was also found in Villa Rotonda and to a lesser extent in the Maison Louise Carre. The generated results also all show traces of noise from the video-guided diffusion process, resulting in geometry features that don’t relate to either the prompt or the input model. The colouring of the generated models at times suggests further architectural ideas to augment the material composition of the input houses such as the added concrete walls (green) for the House at Mols Hills and the inserted wood roof (orange) in the Maison Louise Carre.

**Figure 7.**
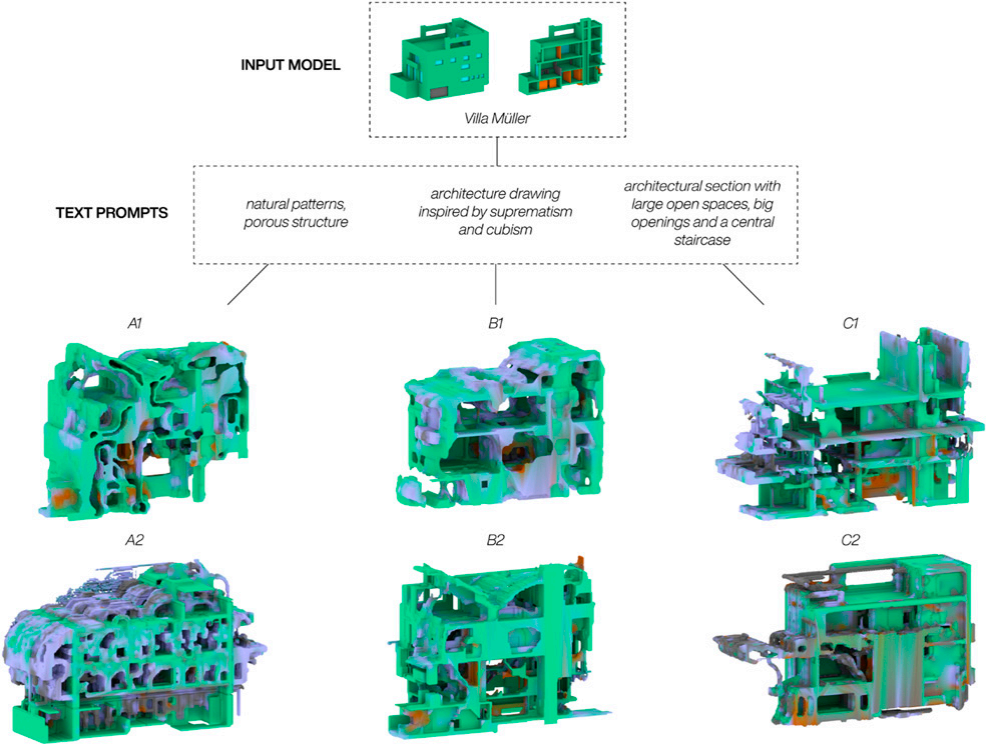
Text-prompts results: The same input model with three different prompts.

For the second approach, three different prompt strategies were applied to the same 3D model from the dataset, Villa Müller by Adolf Loos. The intent was to determine the degree to which the diffusion process could react to these strategies and propose design suggestions. Figure 7 highlights the results, depicting two outcomes from each of the deployed strategies. Each generated 3D model is cut in half and visualized through a meshed section model to best depict the transformation. Model A1 and A2 were generated with the prompt natural patterns, porous structure, and the intention was to describe concepts not found within the dataset. The generated models showcase an intricate and organic interior with clear references to the prompt. Though their interiors might be hard to inhabit as is, both models depict spatial relations not found within the Dreambooth training data, demonstrating the method’s ability to link the style of the custom training with the overall generative capabilities of the SDXL-Turbo model.For Model B1 and B2, where the prompt was architecture drawing inspired by suprematism and cubism, the transformations have a closer relation to the input model interior and are as such easier to imagine as a functioning sequence of architecture spaces. In model B1 this is shown through interconnected rooms with varying heights not unlike the raumplan of Villa Müller, and in model B2 through new roof spaces and a double-height room on the ground level with what could be interpreted as circulation cutting through. The idea behind the prompt was to describe the 2D slices from Villa Müller, generating new design suggestions with a visual similarity to the input model. The last prompting strategy was to write a specific design request to create a targeted architectural transformation. Model C1 and C2 suggest how Villa Müller could look if the request was to create large open spaces, big openings and a central staircase. For C1 the request seems to have similarities to the desired outcome, with large open, partially unobstructed floorplates extending beyond the facade of the input model. The facade is also partially dissolved, creating a lot of openings with what could generously be interpreted as an attempt to implement a central staircase in the middle of the building. Model C2 however indicate very few of these readings, questioning if the relative success of C1 was down to a lucky seed within the diffusion process rather than the network’s ability to pair the very specific text prompt with a meaningful outcome from an architect’s point of view. Other results not shown in this paper have further indicated that this is most likely the case.

#### Image-prompts

**Figure 8.**
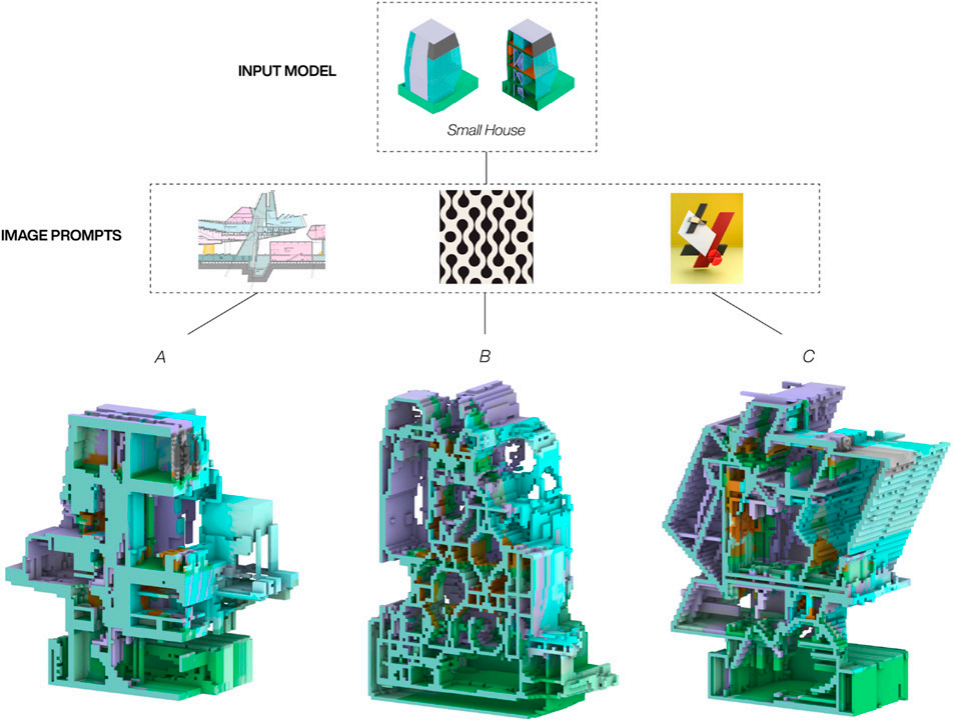
Image-prompts results: The same input model stylised with three different images.

While text prompts describe the desired outcome or transformation in words, image prompts enhance this by adding a visual dimension, directly associating information with specific locations in a 2D space. This makes image prompts particularly effective for guiding spatial modifications, as they provide a more intuitive and immediate way to communicate structural or stylistic changes.The evaluation of the image-based prompts was done on a 3D model of the Small House by Kazuyo Sejima and explored its transformation through three different images (see Figure 8). The first image depicts a cropped architectural section drawing of Volcania by Hans Hollein and model A shows a cut section model of the generated house it produced. While the resulting 3D model kept the overall proportions of the Small House, maintaining the central circulation core, the outcome also breaks with its simple silhouette, introducing a series of terraces, protrusions and cutout in the original geometry. This seems partially reminiscent of its image prompt, which also has a silhouette with similar features. The second and the third image shows a seamless 2D geometry pattern and an abstract spatial composition inspired by suprematist art – and model B and C illustrate how these two image-prompts respectively transformed the input house. In both cases the input images influence is easily readable in the generated 3D transformation. Whereas the seamless 2D pattern is only directly visible in the bottom half of model B, with other parts only subtlety affected such as the new vaulted roofs, the composition of the third image overwrote the entire form of the Small House in model C – transforming its silhouette and internal division to align with the figures in its image-prompt. The reason for this could come from a compositional and stylistic similarity between the third image and the sliced image sequence of the input model, with both having coloured figures in a vertically arranged configuration, while being framed by a simplistic background. As the settings for the video-guided diffusion process was kept identical for the generation of all models, the results demonstrate how different images can successfully be used to initiate a variety of 3D transformation of the same input model. The outcomes also highlight how the material-based colour information is mostly perceived thanks to the Dreambooth training, even though the input images introduce their own colours into the process.

#### Video-prompts

**Figure 9.**
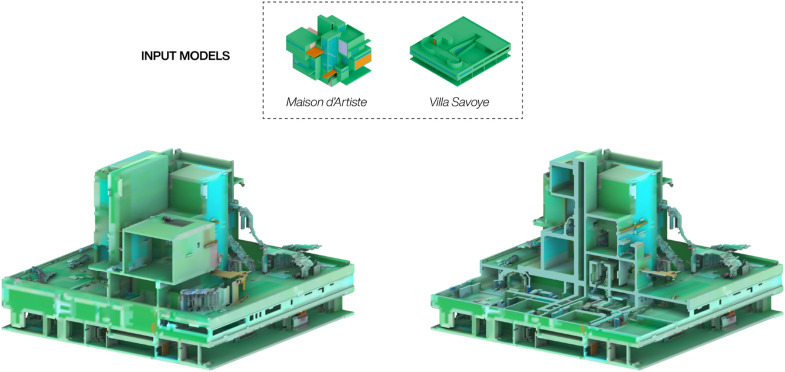
Video-prompt result: Two input models are blended during through their sliced image sequences.

Video-prompting works similarly to image-based prompting at the generation of a single image. But where an image-based prompt maintains the same ‘style’ image throughout the generation process of an entire image sequence, a video prompt updates the ‘style’ image at every frame, hence enabling the transformation of one video with another – or in our case, blending two 3D models. Figure 9 shows an outcome from blending a 3D model of Maison d’Artiste by Theo van Doesburg with one of the Villa Savoye by Le Corbusier. While the video-diffusion settings were identical to the previous evaluation results, each model furthermore had an equal weight within the process, resulting in a generated model that tries to spatially negotiate the insertion of the two models within the same space. In this case no prompt was used beyond the key phrase to initiate visual style of the trained Dreambooth checkpoint, but the setup could be combined with further text- and image-prompts. The generated geometry highlights the potential of video-prompts to hybridise existing 3D models, which can also act as inputs for other video-diffusion processes.

### Comparison

**Figure 10.**
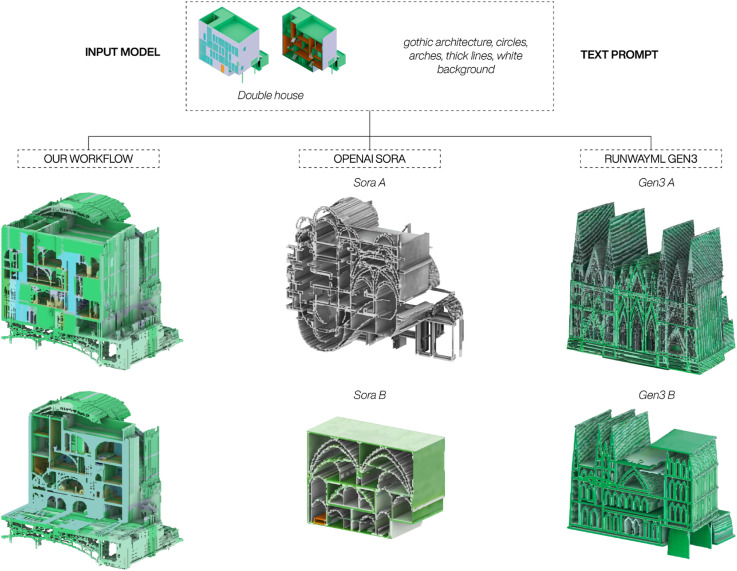
Comparison of our workflow to mainstream video-guided diffusion platforms. During the generation of Sora A and Gen3 A the text-prompt had a higher priority, whereas Sora B and Gen3 B prioritised the input model.

Video-diffusion is developing rapidly, and several existing platforms provide an easy-to-use interface for transforming videos through texts, images and other videos - among these choose the two most prominent RunwayMLs GEN3^42^ and OpenAIs Sora.^43^ To compare our workflow with these state of the art platforms, we use the same input model, the Double House by MVRDV, and the same text prompt: gothic architecture, circles, arches, thick lines, white background. Figure 10 shows a comparison of how the three different frameworks manage to generate new 3D models which both adhere to the prompt and the input model.

For Sora A and Gen3 A the prompt had a higher weight than the input model, whereas the opposite was true for Sora B and Gen3 B. Compared to our workflow, both Sora and Gen3 struggle to generate a complex 3D model beyond a simple 2D extrusion of one of the initial images. Though both Sora and Gen3 are highly capable frameworks, the results highlight the importance of both the custom training through Dreambooth and the advanced adjustments allowed by ControlNet to successfully use the sliced image sequences in the generation of new 3D models.

### Application in design seminar

**Figure 11.**
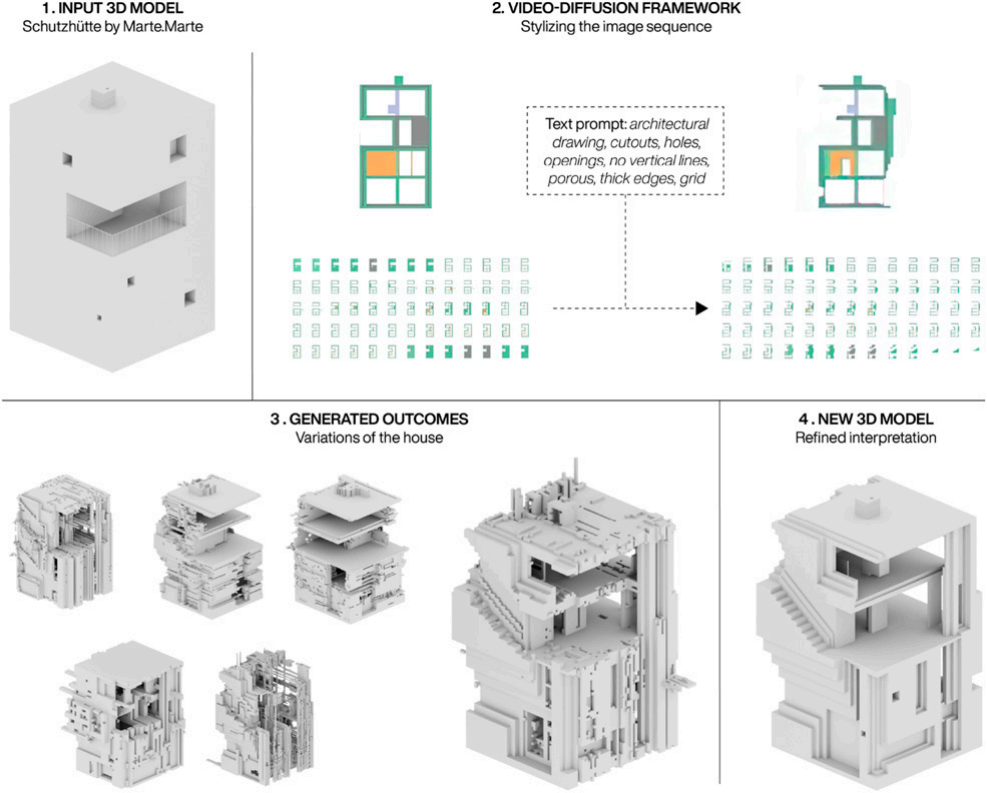
Transformation of an existing 3D house model through the video-diffusion framework. Six generated outcomes are shown and one is selected for refinement through a geometric clean-up process and interpretation. Design work by Maximilian Zörer.

The frameworks potential was tested in a bachelor design studio to explore how it could aid the ideation and design process. Initially, it was introduced through an introduction task, where students were asked to transform an existing 3D model of a house with the workflow. Figure 11 shows one of the more successful outcomes from this process. Here a student used it to transform a 3D model of the Schutzhütte from Marte. Marte Architects, in order to create a more open and extrovert house.The same text prompt was used with different seeds, steps, CFG and strength scheduling values in order to explore different generated outcomes, while the segmentation model was kept at a weight of 2. At the end a desired outcome was chosen and refined through both automated and manual processes to clean up noisy surfaces. Lastly, the student interpreted elements from the generated geometry and tried to translate them into meaningful design features, such as the diagonal extrusion on the facade which was turned into a staircase, in order to create a new 3D model of a house. Though the process in this case ignored the colour information within the images when reconstructing the image sequences, the final outcome depicts a new house which aligns with the students original intention for the transformation. The new 3D model has the same dimensions as the original house, but appears extroverted in the top half, where even the circulation has been pushed onto the facade. Though some of this is down to the interpretation of the student, the process highlights how the framework can function as a new tool for ideating on existing 3D geometries.

**Figure 12.**
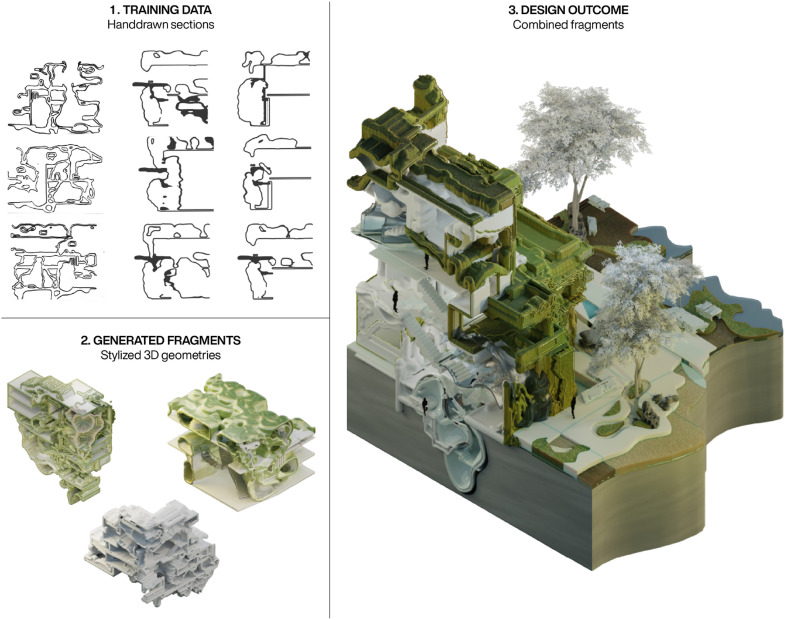
An adaption of the video-diffusion framework with a custom 2D dataset was used to stylize rough 3D fragments, before they were combined into a final design outcome. Design work by Jolina Thome.

In the second scenario, the workflow was adapted to support a student in developing the final design for a bachelor project. Figure 12 highlights key excerpts from this process.Instead of engaging with 3D models using text-, image- or video-prompts, a custom-trained Dreambooth checkpoint was created. This checkpoint was developed using a dataset of hand-drawn sections inspired by organic cell structures. By fine-tuning the SDXL-Turbo model,^35^ the system learned the visual language of the dataset, which was then linked to a unique keyphrase - enabling it to be used as the sole prompt in a video-diffusion process. With this setup, simple and rough 3D model fragments were transformed into stylized geometries that reflected the visual language in the hand-drawn dataset. Finally, the generated fragments were refined and combined, either fully or partially, to create the final design of the bachelor project. The outcome demonstrates how the video-diffusion process can be customised to achieve a targeted outcome, enabling a stylistic transfer of 2D visual aesthetic into three dimensions.

## Discussion

The findings indicate that integrating video-guided diffusion with image sequences from architectural models enables designers to experiment with and apply diverse stylistic influences to 3D models. The presented workflow demonstrates, how this process affects both the exterior and the interior of the model, thereby generating design suggestions that go beyond the low-resolution boundary shapes of current text-to-3D applications. Unlike most existing 3D generative approaches in architecture, the resulting 3D models can also incorporate additional design information through colour-encoding. This means that the workflow in principle can work with databases of BIM models or preliminary massing models with or without labels. In our case, the colours represented the primary materials of the various building elements within each house. Though we were successful in bringing this information into the workflow, the results also showed that not all new design suggestions and transformations beyond the input models were coupled with a clear material label. This leads to several material gradients in the outcomes, which indicates that the encoding of other information such as programmatic functions, where it’s easier to imagine mixes, might be more beneficial for early-stage design ideation.

We also observed that pairing custom training data through Dreambooth with the base Stable Diffusion model (SDXL-Turbo) was beneficial both for implementing design concepts from outside the basic training data and for biasing outputs towards its aesthetics. As a result, the setup can produce a large variety of outcomes which still adhere to the visual style and scale of the used 3D dataset. The high resolution of the generated models meant that the design outcomes were not restricted to broad morphological features but also included fine local details, further enhancing their potential ideation utility in the design process. Additionally, because the 3D-to-2D encoding yields planar image sequences without perspective or shading, the video-guided diffusion appeared most precise when prompts described concepts easily represented within this visual setup such as curves, cubism, cells, diagonals etc. Compared with established video-diffusion platforms from RunwayML and Open AI, the presented workflow also proved superior at generating spatially complex 3D models, validating the benefit of the custom trained Dreambooth checkpoint and the enhanced controls provided by ControlNet to guide the diffusion process. Additionally, the application of the framework within a bachelor design studio, showed two examples of how the setup could successfully aid designers. In both cases the setup enabled a transformation from one 3D model to a new 3D models based on a design intent. Though the application of the workflow within a design studio explored ways to post-process and optimise the generated ’concept clouds’/3D models through voxelisation and meshing with Signed Distance Field algorithms, it remains unexplored beyond those methods. Therefore, future research in these areas may offer both quantitative and qualitative improvements, further clarifying and interpreting the generated concept clouds usability within a design context.

### Limitations

With our current setup, generating a result on a computer with a NVIDIA GeForce RTX 3080 Ti takes approximately three to 6 minutes. While the speed of 2D diffusion models has increased a lot over the last 2 years, but the generation of 200-400 images is still a relatively slow process. This means that the creative ideation and usefulness of the outlined approach could be increased enormously with further advances in image and video AI generation or access to cloud based GPUs. Though the resolution of the generated models is high, thin elements still tend to disappear. Within our 3D dataset, this is especially happening to glass elements, meaning that a lot of the current openings in the results should have been closed. On the other hand, the lack of glass is beneficial for the reading of the spatial organisation, and interior-exterior relations of the produced models. An ideal solution would be to remove elements relating to glass and openings such as doors within the 3D dataset to improve the readability and consistency of the outcomes or find ways to further increase the resolution without exponentially increasing the generation time. Additionally, the frameworks’ emphasis on section drawings could be enhanced by incorporating sliced image sequences along different vectors. This would allow plan drawings to integrate into an iterative generative process, enabling more comprehensive stylistic transformations of 3D models.

Although the presented video-diffusion pipeline yields visually coherent full-model transformations in a single pass, it can not facilitate a successive refinement from shell to structure to interior that phase-based related methods such as ArchiDiff^44^ inherently support. In professional practice, architects typically demand a fine-grained control over each design phase.^45^ Future work could therefore explore a hybrid approach that first apply diffusion to exterior slices, then run a dedicated interior-focused stage, thereby reconciling the immediacy and visual novelty of this papers pipeline with the detailed oversight of phase-based development.

## Conclusion

The relationship between AI technologies and architectural design processes is expected to continue evolving in the coming years. While the technical foundations of this study are advancing rapidly due to developments in video diffusion models such as OpenAIs Sora,^43^ this research emphasises a conceptual framework for 3D generation that departs from most existing image-based methods. By generating a 3D model through a diffused tomographic scan, the methodology offers an inside-out approach to exploring new design concepts. This method can suggest complex 3D interior-exterior relations, highlighting an important advantage over the dominating outside-in approach. Where the outside-in approach strives to generate a set of perspectival images from various viewpoints to predict a 3D boundary shape similar to photogrammetry, our approach prioritizes the dialogue between interior and exterior shapes, enhancing its usefulness for ideation with architectural 3D models.

Beyond the presented framework in this paper, another promising avenue of exploration involves the development of 3D generative models trained directly on geometric representations of architectural objects, such as voxel grids, rather than relying on 2D images, as is common in existing 3D shape generation methods.^46^ Such a data-driven approach, even in its basic form, would enable the learning of 3D interior-exterior relations from a large collection of 3D architectural objects, while facilitating the synthesis of a diverse set of high-quality, structured new designs inspired by the existing ones.
